# Assessing the Influence of Screw Orientation on Fracture Fixation of the Proximal Humerus Using Finite Element Informed Surrogate Modeling

**DOI:** 10.1002/cnm.70060

**Published:** 2025-07-04

**Authors:** Daniela Mini, Karen J. Reynolds, Mark Taylor

**Affiliations:** ^1^ Medical Device Research Institute College of Science and Engineering, Flinders University Adelaide South Australia Australia

**Keywords:** finite element analysis, locking plate fixation, neural networks, proximal humerus fracture, screw direction, surrogate modeling

## Abstract

The management of proximal humeral fractures is challenging, and fixation plates often show a high failure rate. However, new fixation plates with variable angle screws could be beneficial. Finite element (FE) studies have shown some benefits of plates with variable angle screws, but not all possible combinations have been explored, and hence worst and optimal scenarios have not been identified. The full exploration of the solution space is not possible using FE techniques due to the computational expense; therefore, a more computationally affordable technique is needed. This study aimed to develop adaptive neural network (ANN) models that can predict the likelihood of a screw collision and the level of strain on the humeral bone when the orientation of the screws is changed. ANN models were trained using input and output data from FE simulations with varying screw angles, developed on a single subject with a two‐part fracture in the proximal humerus. Training sets of different sizes were developed to determine the quantity of data required for an accurate model. Firstly, the ANNs were used to make predictions of results from FE unseen data, showing an 84.4% accuracy for the prediction of screw collision and good correlation (*R* ^2^ = 0.99) and low levels of error (RMSE = 0.65%–5.49% strain) for the prediction of bone strain. The ANNs were used to make predictions of a full factorial scenario, showing that the variation of the orientation of the screw in the calcar region has the greatest impact on the bone strain around all screws.

## Graphical abstract

In this work, proximal screw orientation was varied to generate finite element (FE) models of the humerus with a fracture fixation plate. The FE data were used to train an adaptive neural network (ANN) to predict whether variations in the proximal‐distal and anterior–posterior orientations of the screws would impact bone strain. An accurate prediction of the influence of screw direction on bone strain was achieved by the integration of FE data and ANN approach, providing a computationally efficient tool.

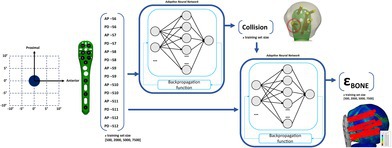

## 1 Introduction

Fractures of the proximal humerus are one of the most frequent fractures in older persons [1], accounting for around 10% of all fractures, with a higher incidence for women > 65 years old and predicted to increase with the increase of the older population [2, 3]. Up to 30% of these fractures are treated surgically in Australia and the most common surgical procedure is the use of a fracture fixation plate [2]. Fracture healing is not always achieved, with a reported failure rate of up to 35% [4]. The design of fracture fixation plates has changed in the past decades intending to decrease the failure rate, and recently fracture fixation plates incorporating variable angle screws have been introduced. These variable angle screws can improve the stability of the fixation by adapting to various fracture patterns and variations in bone mineral density, ensuring a more tailored and effective process to bone healing [5]. In vitro testing is the gold standard for evaluating implant fixation biomechanics [6], but it becomes impractical due to the high number of parameters to investigate and cannot explore the impact of different screw configurations for a specific subject. On the other hand, finite element (FE) techniques have been used to explore the impact of different variables on the bone strain and implant stress for a fracture fixation plate [7], such as the position of the plate [8], screw length [9], bone quality [10, 11], the number of screws in the head of the humerus [10, 11] and their orientation in space [12, 13, 14, 15]. However, these studies have been conducted on a limited number of configurations ([9, 10], 2019b; [11, 12, 14, 15, 16, 17]), with simulation numbers ranging from a few hundred ([10], 2019b; [11, 12, 15, 17]) to a few thousand [9, 14, 16].

Only a few studies have investigated the impact of screw orientation on predicting mechanical failure of the locking plate fixation [12, 13, 14, 15]. Jabran's study focused only on the variation of the orientation of two screws on a single subject, generating 538 configurations [12], and Mischler and Schader's studies were both conducted on a group of 19 subjects but varying the screw orientation of the six proximal screws only a one at a time, for a total of 88 configurations per sample [14, 15]. Additionally, in a more recent study, the Mischler group conducted experimental validation confirming that modifying the direction of specific screws led to improvement in preventing cutout failure when compared to using the standard fracture fixation plate [13]. The Jabran study only focused on the angle variations of two screws, while the first Mischler and Schader studies explored a considerably reduced number of screw angle combinations. Indeed, once a large number of simulations need to be investigated, FE analysis becomes too time‐expensive, and a more efficient computational technique is needed. Surrogate models can be used to make estimations of a larger number of configurations in a quicker way. Surrogate models aim to make a prediction of the fitness function of a complex problem with the use of input and output data [18], reducing the computational effort. Specifically for biomechanical applications, Kriging [19, 20], adaptive neural network (ANN) [21, 22] and Gaussian process (GP) [23, 24] based models are some examples of surrogate models that have been used with a combination of FE data to describe problems with hip and knee implants. ANN, which consists of a network of interconnected neurons that exchange information with one another, has been shown to be a precise and effective technique [22]. Recently, our research group successfully developed an FE‐informed ANN model to accurately predict bone strain with varying screw lengths for a proximal humeral fracture with a fracture fixation plate. The results of this study have demonstrated a high level of accuracy and have shown great promise for the application of ANN models in this field [21]. The aim of the current study is to develop an FE‐informed ANN model to analyze the effect of screw orientations on bone strain for a fracture fixation plate implanted in a humeral head, reducing computational time and exploring a wide range of possible configurations. Moreover, to enhance computational efficiency, an additional ANN model was created to eliminate configurations in which two or more screws may physically intersect, classifying them as collisions. A hypothesis of this study is that the variation of the angle of the calcar screw has a higher impact than the other screws, as has been shown in other studies [14].

## 2 Methods

In order to investigate how screw orientation affects bone strain, various FE models were generated by changing the direction of the screws in the proximal‐distal and anterior–posterior directions. The input and output data from these models were then used to train different ANN models, and their accuracy was analyzed. More specifically, a single ANN model was developed to predict bone strain around the screws, while two separate ANNs were developed to together detect potential screw intersections, referred to as collisions, and to assess the bone strain around the screws.

### 2.1 FE Model

**FIGURE 1:**
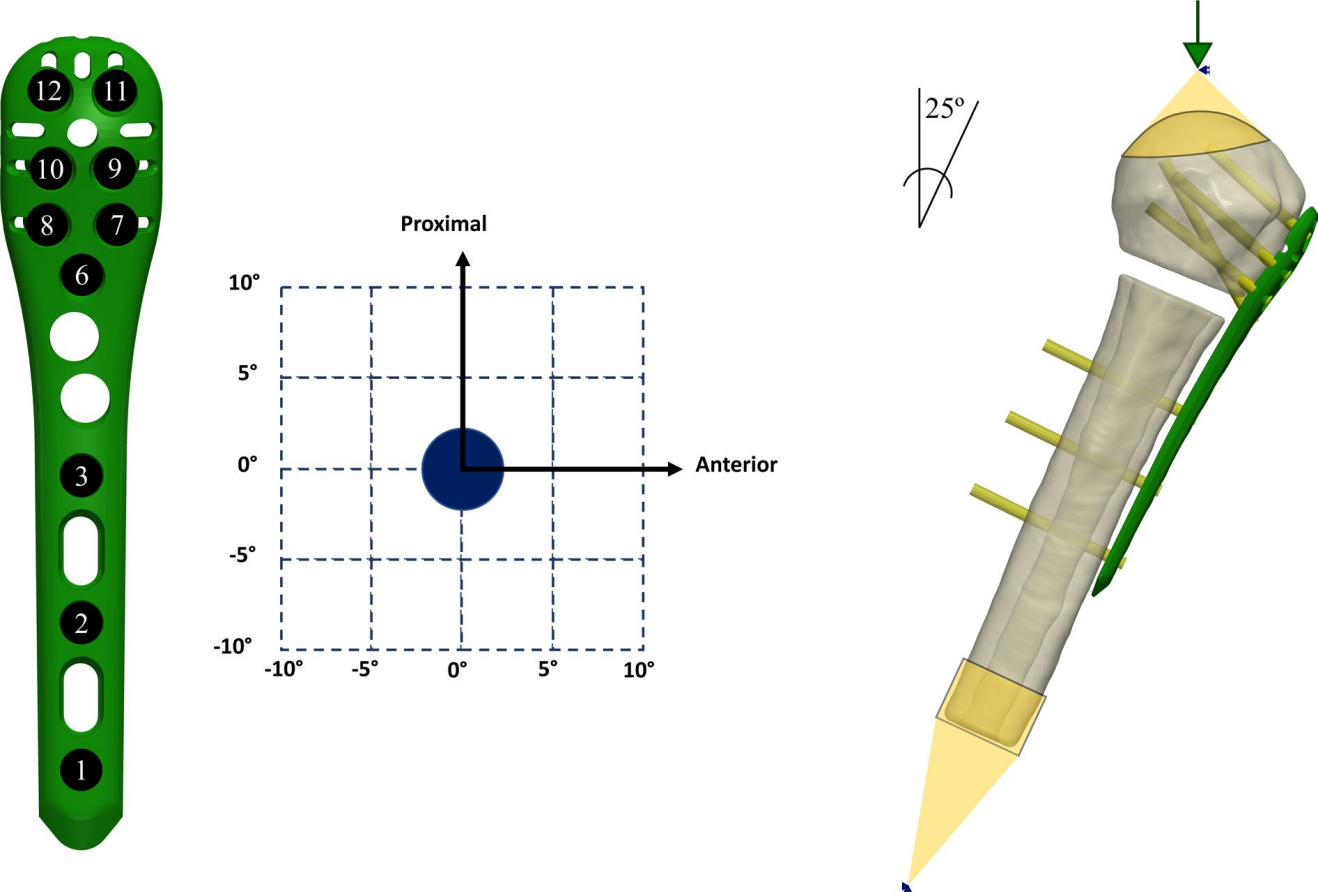
Numerations of the screws used for this study (left). Possible orientation of the screw tips in distal‐proximal and posterior–anterior direction (center). The head of each screw was fixed in the neutral position (0°–0°), and the tip of the screw was able to move from the neutral position with increments of 5° in the distal‐proximal and posterior–anterior direction. Loading and boundary conditions (right). Image from Reference [21].

For the generation of the FE analysis, one CT image of a cadaver of a 61‐year‐old female donor was collected from the New Mexico Decedent Image Database (NMDID) [25]. The right humerus was manually segmented using Simpleware software (Version U‐2022.12; Synopsys Inc., Mountain View, USA) and the shaft was cut at 160 mm length from the humeral head. A single cut with a 5 mm gap at the surgical neck was virtually performed, representing a single two‐part proximal humeral fracture AO/OTA 11‐A2.1 [26]. The reason for choosing a two‐part fracture was to create a simplified model with reduced computational cost. Additionally, this choice provided consistency with our previously published study. The bone fracture was virtually fixed using a fracture fixation plate (Austofix, Adelaide, Australia) (Figure 1). The plate was positioned 4 mm posterior to the bicipital groove and 7 mm distal to the top of the greater tubercle, and it was secured in place using seven proximal screws and three distal screws, each represented as a cylinder of 3 mm diameter. This configuration followed Austofix guidelines and aligned with the screw dimensions used in previous finite element studies. Moreover, according to Fletcher et al., a comparable proximal humeral plate demonstrated lower strain values when implanted with more than six screws. Therefore, this study uses six proximal screws along with a single calcar screw, resulting in a total of seven proximal screws. The orientation of the seven proximal screws was varied in the proximal‐distal and anterior–posterior direction from the neutral position (0°–0°) (Figure 1), which was defined as the standard configuration of the screws, determined by Austofix guidelines. The length of each of the screws was varied in order to have a Tip to Joint (TJD) distance of 8 mm [9], defined as the distance between the tip of the screws and the glenohumeral joint. All materials were defined as linear elastic with a Poisson's ratio of 0.3. The material properties of the bone were defined as heterogenous, in which the bone mineral density (BMD) was derived from the CT image using the phantomless calibration methodology proposed by Eggermont et al. [27]. The elastic modulus was converted from the local BMD using the Morgan et al. [28] equation: $E\;\left(\mathrm{MPa}\right)=6850\rho_{\mathrm{app}}^{1.49}$. The mean value of BMD in the humeral head had a value of 66.39 mgHA/cm^3^, comparable to the range values found in the literature [29, 30]. The screws and plate were defined as titanium alloy, with a Young's modulus of 105 GPa. Synopsys' Simpleware FE module was used to generate a mesh of the model with linear tetrahedral elements (C3D4), having an element edge length between 1 and 0.5 mm. Bone‐screw and screw‐plate interfaces were set as tied contact. The models were subjected to a vertical force, reproducing the axial bending scenario according to Röderer et al. experimental study [31]. In agreement with Bergmann's clinical study [32], the model geometry was rotated by 25° around the anterior–posterior axis of the humerus. An external point that represented the midpoint between the condyles of the distal humerus was fixed in all directions and connected to the nodes of the distal portion of the humerus. A second external point, located at 1 mm distance from the surface of the humeral head along the axis connecting the center of the humeral head to the center of the circular region, was linked to a circular region with a diameter of 20 mm located on the humeral head. A vertical force of 100 N was applied on the second external point, free to move only in a vertical direction (Figure 1) and no rotations were allowed. The magnitude and orientation of the applied force were consistent with those reported in the first validated study by Varga et al., and they also align with the loading conditions of our previously published research. The minimum principal strain of the bone was evaluated at the surface surrounding the proximal screws, as this has been shown to be a surrogate parameter of failure [33]. Indeed, Varga's study demonstrated that the minimal principal strain around the screws performs as an sufficient predictor for cutout failure of the implant. A standard implicit static analysis of the models was run in ABAQUS (Version 6.14–3, Dassault Systèmes, Vélizy‐Villacoublay, France). An automated workflow was developed using Matlab, as a way of speeding up the FE model generation, solution and post‐processing pipeline.

Figure 1 Numerations of the screws used for this study (left). Possible orientation of the screw tips in distal‐proximal and posterior–anterior direction (center). The head of each screw was fixed in the neutral position (0°–0°), and the tip of the screw was able to move from the neutral position with increments of 5° in the distal‐proximal and posterior–anterior direction. Loading and boundary conditions (right), image from [21].

The process included the generation of the models with the meshing process in Simpleware, the setting of the boundary and loading conditions in Matlab (Mathworks, Natick, MA, USA), the running and output analysis using Abaqus, and the post‐processing of the data. Moreover, an automated process of selecting the screw length, imposing a TJD of 8 mm [9] and detecting any screw collision in the model was developed and integrated into the workflow.

### 2.2 Dataset Development

**FIGURE 2:**
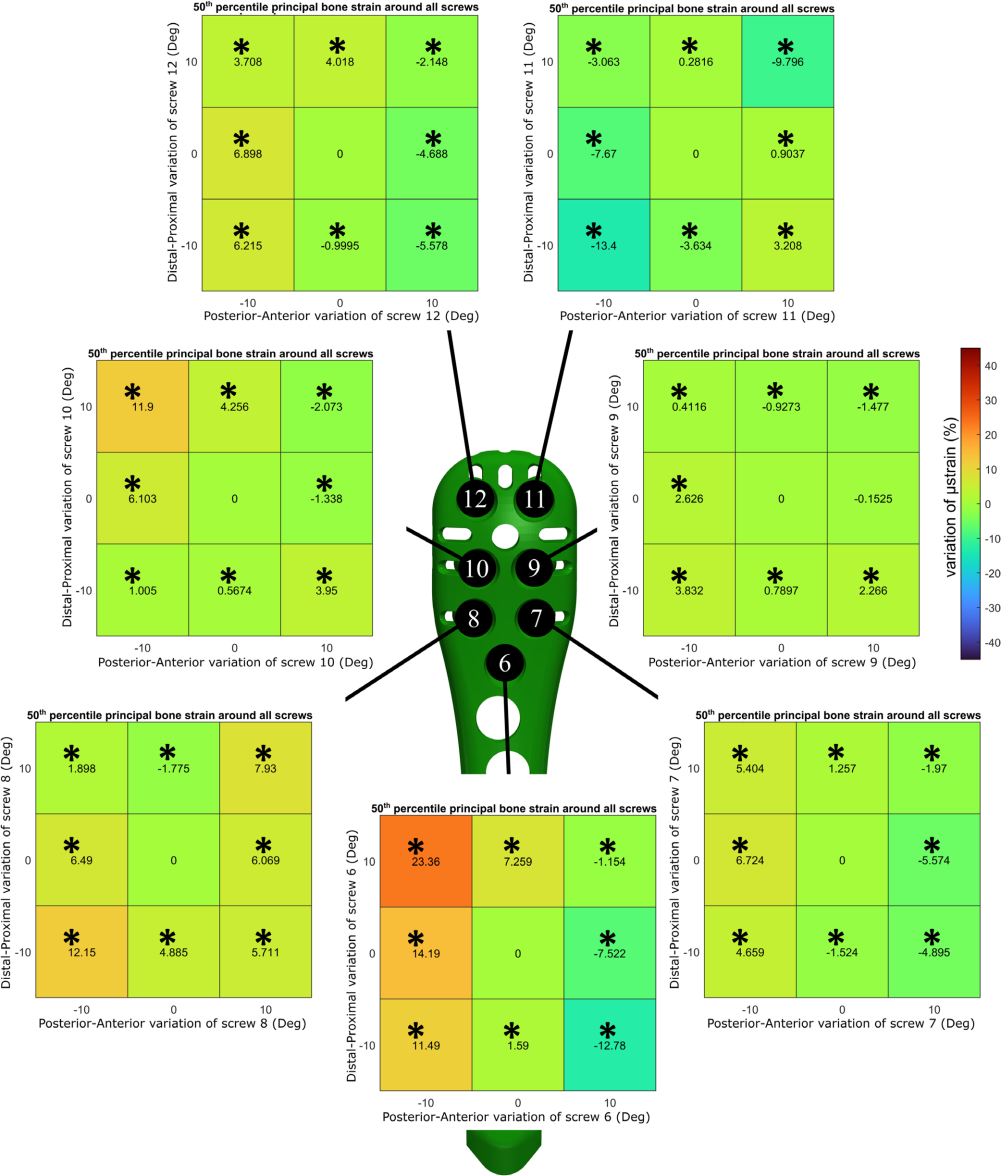
Prediction of median principal bone strain around all the screws. Each heatmap represent the percentage of variation of strain for each screw from its neutral position (* indicates *p* < 0.001).

The proximal‐distal and anterior–posterior orientation of the seven proximal screws was varied from the standard position, which corresponds to the original position of screws in the plate configuration (0°–0°). In particular, the location of the head of each screw was fixed and the position of their tips was changed from the conventional position. For both directions, the tip was positioned from a range of −10° and 10°, varying with an increment of 5°. Therefore, the overall number of potential configurations can be expressed as (5 × 5)^7^, wherein ‘5’ represents the possible number of positions in both proximal‐distal and anterior–posterior orientations, and ‘7’ represents the number of screws allowed to adjust their orientation. The orientation of the screws was adjusted in combinations. To reduce this number, three training sets of different sizes were developed with a Latin Hypercube sampling technique, generating training sets of 500, 2000, and 5000 FE models. Some simulations failed due to screws colliding when changing direction, resulting in 92, 370, and 879 successful simulations for each group, respectively. In addition, a final training set size of 7500 was considered as the sum of all the training sets, with a total of successful simulations of 1341. To test the surrogate models once developed, a testing set size of 500 simulations was created, which resulted in 91 successful simulations after removing invalid models due to screw collisions. The training datasets were used to develop and train three different ANNs, one for the detection of screw collision and the other two for the prediction of the principal minimum strain of the bone at the strain surface. All the ANNs were developed using the Neural Network toolbox in Matlab (Mathworks, Natick, MA, USA). (Figure 2).

#### 2.2.1 ANN for Detection of Screw Collision

The first ANN developed was a classification type network, developed in order to detect if any screw of a model was colliding with any others. A collision was defined as any contact occurring between screws. The network used was structured with a single hidden layer composed of 25 neurons and one linear output layer, and the training algorithm used was a Levenberg‐Marquard backpropagation function. The network was fed with the information of the anterior–posterior and proximal‐distal position of each screw, for a total of 14 inputs. The output information was a binary representing the collision or non‐collision of the screws. This ANN will be addressed as ANN_collision_.

#### 2.2.2 ANN for Prediction of Bone Strain

The second and the third ANNs were composed of two hidden layers, with 10 and 5 nodes respectively, and one linear output layer. The input used to train the second model were the orientation of the screws and the information of screw collision, for a total of 16 inputs. The outputs used were the medial (50th) and 90th percentile of the minimal principal strain of the bone at the surface of the screws. In particular, ANN models were developed to predict the minimal principal strain of the bone around each single screw and around all the screws. A Bayesian regularization backpropagation function was used to train the ANNs. This ANN will be addressed as ANN_strain_16_.

The third ANN was developed for the prediction of bone strain around the screws. The properties were the same as the ANN_strain_16_, with the difference in the number of input data used to feed the model. Only information of screw orientation was used, without the information of collision of the screws, for a total of 14 inputs. This ANN will be addressed as ANN_strain_14_.

The development of these two distinct ANNs aimed to determine whether incorporating data on screw collisions could enhance the accuracy of predicting bone strain around the screws.

### 2.3 Assessment of ANN

During the training process of all the ANNs, the training sets data were divided into 80% for the training, 10% for the validation and 10% for the test. Therefore, as the training samples were chosen randomly at the beginning of the training process, the training process of each case was conducted 100 times, generating 100 unique ANN_collision_, ANN_strain_14_ and ANN_strain_16_ models, in order to assess the robustness of the networks. The value of 100 was chosen arbitrarily, but it was also previously referenced in the study by Taylor et al. [22]. Those models were then used to make predictions of the 91 unseen cases previously generated for the testing set, and the accuracy of their prediction was evaluated. In particular, for the ANN_collision_ the accuracy was defined by analyzing the percentage of true prediction of the collision. For the ANN_strain_16_ and ANN_strain_14_, a regression analysis was conducted reporting a coefficient of determination (*R* ^2^), regression slope and root mean square error (RMSE), to assess the quality of the predictions of the ANNs of the minimal principal strain of the bone around all the screws and the single screws.

### 2.4 Analysis of ANN Predictions

After the influence of the training set size was assessed, the best trained ANN_collision_, ANN_strain_14_ and ANN_strain_16_ were used to make predictions of a reduced full factorial scenario, in which screws were able to vary in proximal‐distal and anterior–posterior direction of only +10° and −10° from the neutral position. For this setup, the total number of possible configurations was 9^7^. An analysis was conducted to compare the accuracy of predictions between the use of ANN_collision_ combined with ANN_strain_16_ and ANN_strain_14_. To do so, an additional testing set of 500 simulations was defined through Latin Hypercube sampling method in which the TJD of the seven proximal screws had randomly distributed values of −10°, 0°, and 10°, having a successful 96 simulations without collision. A two‐sample t‐test was conducted between the FE results and their predictions using ANN_collision_ with ANN_strain_16_, and their predictions using only ANN_strain_14_.

Lastly, a comparison was made between the 50th and 90th percentile principal bone strain around the calcar screw (Screw 6) using the results of the full factorial generated with the ANN_collision_ with ANN_strain_16_, and the 50th and 90th percentile principal bone strain around the calcar screw (Screw 6) obtained from the training sets of 500, 2000, 5000, and 7500 FE models. This comparison was focused on the calcar screw as it has been shown to be highly influential [14]. The purpose of this comparison was to determine whether there was a difference in the range of strain distribution between the predictions made on the full factorial space and the training sets. To test this, a t‐test was conducted with a statistical significance level of *p* < 0.001.

## 3 Results

### 3.1 Training of ANN Model

A total of 1528 FE simulations were run and each one took between 15 and 20 min, from the generation of the mesh to post‐processing the results. The training time of each ANN was a few minutes and, once trained, the ANNs prediction time of new configurations was just a few seconds.

Firstly, the influence of the training set size was assessed for ANN_collision_ and ANN_strain_16_. For the prediction of collision, a percentage of true and false prediction was reported, showing a minor improvement from the smallest to the largest training set, with a true best prediction from 82.6% to 84.4% (Table 1).

**TABLE 1:** Performance of the ANN_collision_ on the testing set of 91 successful simulations for the prediction of collision of the screws.

Training set size (reduced size)	Prediction of screw collision	
	% Prediction	% Error
500 (92)	82.60 (79.80)	17.40 (20.19)
2000 (370)	83.00 (80.75)	17.00 (19.25)
5000 (879)	83.40 (81.19)	16.60 (18.80)
7500 (1341)	84.40 (81.75)	15.60 (18.24)

*Note:* The influence of the training set size is shown. Results are displayed for the model with the best accuracy, while the average of 100 ANN models is shown in brackets.

Regarding the prediction of principal bone strain around the screws, the influence of the training set size was assessed on the ANN_strain_16_ predicting the 50th and the 90th percentile principal bone strain around the screw surface. The ANN_strain_16_ demonstrated an improvement in accuracy using the larger training set size of 7500, reaching an *R* ^2^ value of 0.98 and a RMSE of 129.30 μstrain for the prediction of 50th percentile principal strain, and an *R* ^2^ value of 0.99 and a RMSE of 129.26 μstrain for the prediction of 90th percentile principal strain (Table 2). Results of 50th and the 90th percentile principal bone strain predictions from the ANN_strain_14_ were reported as well, showing respectively a best *R* ^2^ of 0.91 and 0.92 and lowest RMSE of 95.40 and 306.35 μstrain (Table 3).

**TABLE 2:** Performance of the ANN_strain_16_ on the testing set of 91 successful simulations for the prediction of bone principal strain around the screws.

Training set size (reduced size)	Median min principal strain				90th min principal strain			
	*R* ^2^	Slope	RMSE, μstrain	RMSE, % strain	*R* ^2^	Slope	RMSE, μstrain	RMSE, % strain
500 (92)	0.97 (0.97)	0.96 (0.95)	161.82 (176.75)	4.72 (5.15)	0.97 (0.97)	0.94 (0.93)	449.99 (459.19)	8.06 (8.23)
2000 (370)	0.98 (0.97)	0.98 (0.98)	140.92 (159.50)	4.11 (4.65)	0.99 (0.98)	0.99 (1.00)	209.71 (374.50)	3.76 (6.71)
5000 (879)	0.98 (0.98)	0.98 (0.98)	129.49 (138.18)	3.77 (4.03)	0.99 (0.99)	0.99 (0.99)	160.07 (211.98)	2.87 (3.80)
7500 (1341)	0.98 (0.98)	0.99 (0.99)	129.30 (135.03)	3.77 (3.93)	0.99 (0.99)	1.00 (0.99)	129.26 (168.66)	2.32 (3.02)

*Note:* The influence of the training set size is shown. Results are displayed for the model with the best accuracy, while the average of 100 ANN models is shown in brackets.

**TABLE 3:** Performance of the ANN_strain_14_ on the testing set of 91 successful simulations for the prediction of bone principal strain around the screws.

Training set size (Reduced size)	Median min principal strain				90th min principal strain			
	*R* ^2^	Slope	RMSE, μ strain	RMSE, % strain	*R* ^2^	Slope	RMSE, μstrain	RMSE, % strain
7500 (1341)	0.91 (0.84)	0.90 (0.85)	95.40 (124.116)	2.75 (3.62)	0.92 (0.87)	0.95 (0.92)	306.35 (392.85)	5.49 (7.04)

*Note:* Results are displayed for the model with the best accuracy, while the average of 100 ANN models is shown in brackets.

After having assessed the influence of training set size, additional ANNs were generated and trained using the input and output data from the training set with a size of 7500 to predict the 50th and 90th percentile principal strain around each single screw. The trained ANNs, ANN_strain_14_ and ANN_strain_16_, were used to make predictions of the unseen testing dataset, resulting in a high value of *R* ^2^ and a low level of RMSE for all of them (Table 4, Table 5). In particular, the model ANN_strain_16_ showed a high level of *R* ^2^ (*R* ^2^ > 0.99) and low RMSE, ranging between 30.81 and 66.70 μstrain for the 50th percentile of strain and between 62.17 and 144.30 μstrain for the 90th percentile of strain (Table 4). On the other hand, the model ANN_strain_14_ had a lower accuracy than ANN_strain_16_. For the prediction of the 50th percentile of strain, the *R* ^2^ range was 0.94–0.99 and the RMSE ranged between 69.66 and 163.52 μstrain. The regression with the 90th percentile of strain had an *R* ^2^ value between 0.84 and 0.98, and an RMSE value between 157.45 and 461.81 μstrain (Table 5).

**TABLE 4:** Performance of the ANN_strain_16_ on the testing set of 91 simulations for the prediction of bone strain around each single screw.

	Median min principal strain				90th min principal strain			
	*R* ^2^	Slope	RMSE, μ strain	RMSE, % strain	*R* ^2^	Slope	RMSE, μ strain	RMSE, % strain
Screw6	0.999 (0.999)	1.00 (1.00)	66.70 (89.25)	0.65 (0.88)	0.999 (0.988)	1.00 (1.01)	144.30 (372.83)	0.78 (2.03)
Screw7	0.998 (0.997)	1.00 (1.00)	57.24 (67.08)	1.12 (1.31)	0.997 (0.99)	0.98 (0.99)	205.28 (251.17)	1.31 (1.60)
Screw8	0.996 (0.992)	0.99 (0.99)	41.59 (55.62)	0.99 (1.32)	0.996 (0.994)	1.00 (1.00)	108.09 (143.62)	0.77 (1.02)
Screw9	0.997 (0.996)	1.00 (1.00)	40.09 (48.57)	1.37 (1.65)	0.992 (0.985)	0.99 (0.99)	184.04 (239.23)	2.39 (3.11)
Screw10	0.999 (0.998)	1.00 (1.00)	32.47 (42.06)	0.69 (0.89)	0.998 (0.994)	1.00 (1.00)	77.58 (121.77)	1.01 (1.58)
Screw11	0.999 (0.998)	1.00 (1.01)	24.02 (31.04)	0.83 (1.07)	0.997 (0.993)	1.00 (1.00)	84.16 (115.75)	1.30 (1.78)
Screw12	0.999 (0.999)	1.00 (1.00)	30.81 (37.04)	0.74 (0.89)	0.999 (0.999)	1.00 (1.00)	62.17 (81.87)	0.76 (1.01)

*Note:* Results are displayed for the model with the best accuracy, while the average of 100 ANN models is shown in brackets.

**TABLE 5:** Performance of the ANN_strain_14_ on the testing set of 91 simulations for the prediction of bone strain around each single screw.

	Median min principal strain				90th min principal strain			
	*R* ^2^	Slope	RMSE, μ strain	RMSE, % strain	*R* ^2^	Slope	RMSE, μ strain	RMSE, % strain
Screw6	0.989 (0.973)	1.00 (0.98)	163.52 (237.73)	1.62 (2.35)	0.979 (0.946)	0.97 (0.96)	371.37 (583.09)	2.02 (3.17)
Screw7	0.954 (0.872)	0.93 (0.88)	123.57 (188.60)	2.42 (3.69)	0.970 (0.874)	0.92 (0.86)	461.81 (799.31)	2.95 (5.10)
Screw8	0.969 (0.915)	0.99 (0.90)	102.98 (149.99)	2.44 (3.56)	0.981 (0.946)	0.98 (0.96)	264.68 (388.92)	1.87 (2.75)
Screw9	0.946 (0.904)	0.95 (0.91)	91.422 (118.89)	3.11 (4.05)	0.846 (0.694)	0.88 (0.75)	423.15 (590.49)	5.50 (7.67)
Screw10	0.987 (0.965)	0.97 (0.97)	72.036 (109.05)	1.52 (2.30)	0.973 (0.923)	1.00 (0.95)	174.80 (284.37)	2.28 (3.70)
Screw11	0.979 (0.954)	0.97 (0.96)	63.485 (81.61)	2.20 (2.82)	0.970 (0.943)	0.97 (0.95)	182.96 (241.93)	2.82 (3.37)
Screw12	0.992 (0.981)	1.01 (1.01)	69.669 (94.53)	1.68 (2.28)	0.985 (0.936)	1.02 (0.98)	157.45 (250.67)	1.93 (3.08)

*Note:* Results are displayed for the model with the best accuracy, while the average of 100 ANN models is shown in brackets.

### 3.2 Full Factorial Predictions

Afterwards, the best ANNs trained with 7500 FE data were used to make a prediction of a full factorial scenario, of 9^7^ possible configurations, in which the screw could vary in proximal‐distal and anterior–posterior direction of ±10° from the neutral position. The ANNs were used to make predictions of 50th and 90th percentile principal bone strain around all the screws and around every single screw of a new testing set.

A t‐test was conducted between the FE predictions of an unseen dataset that was set as a control group, their predictions using ANN_collision_ with ANN_strain_16_ and their predictions using only ANN_strain_14_. No significant difference was found between the FE data and the predictions using ANN_collision_ with ANN_strain_16_ (*p* > 0.05). Significant differences (*p* < 0.05) were found between the FE data and the ANN_strain_14_ predictions of 50th percentile principal bone strain around Screw 6, screw7, and screw 8, and 90th percentile bone strain around all the screws, screw6, screw8, screw 9 and screw 10.

**FIGURE 3:**
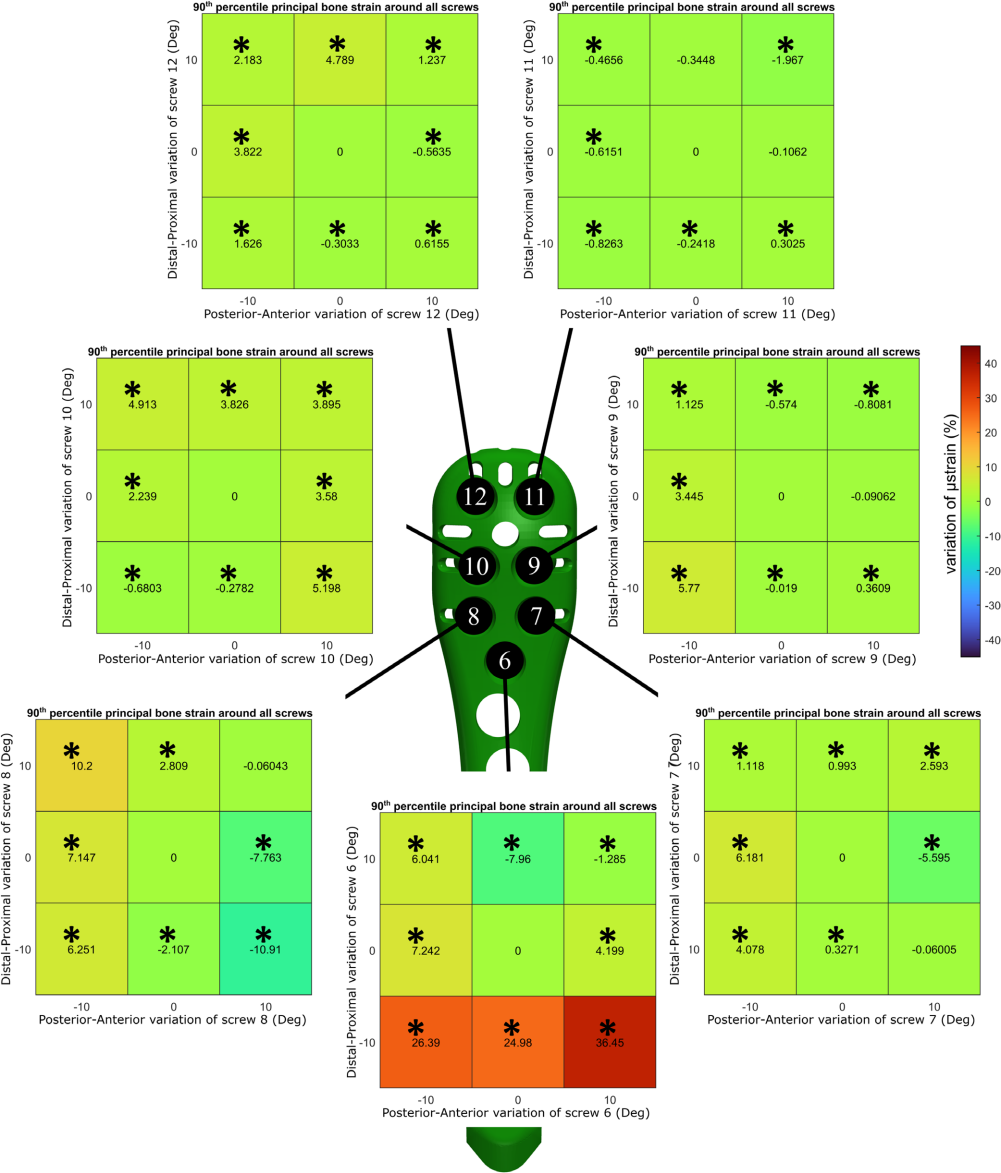
Prediction of 90th percentile principal bone strain around all the screws. Each heatmap represent the percentage of variation of strain for each screw from its neutral position (* indicates *p* < 0.001).

**FIGURE 4:**
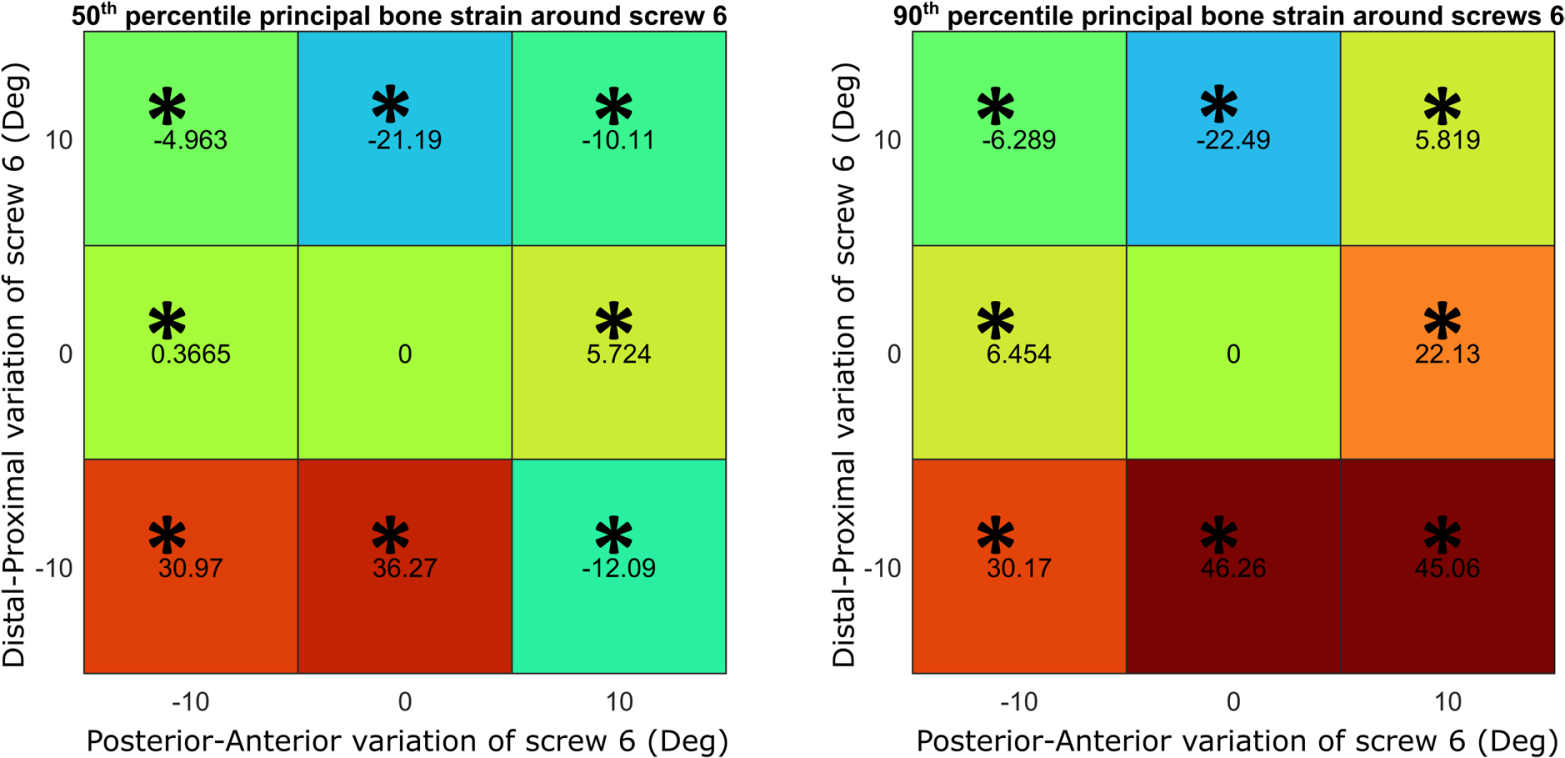
Prediction of 50th and 90th percentile principal bone strain around Screw 6 with the variation of orientation of Screw 6. Each heatmap represent the percentage of variation of strain for each screw from its neutral position (* indicates *p* < 0.001).

Therefore, ANN_collision_ with ANN_strain_16_ was used to make predictions of principal bone strain around the screws of all the 9^7^ possible configurations since there was no statistical difference with the FE results. The influence of the orientation of each screw was assessed, showing a significant difference (*p* < 0.001) in most of the changed positions of their tips from the neutral position (Figures 2, 3). In particular, Screw 6, the one considered the calcar screw, had the biggest impact on the variation of 90th and 50th percentile of bone strain around all the screws showing variations between −12.78% and +36.45% (Figures 2, 3). The influence of the variation of the orientation of Screw 6 on the strain of the bone around itself has been reported, showing variations between −22.49% and +45.06% (Figure 4).

**FIGURE 5:**
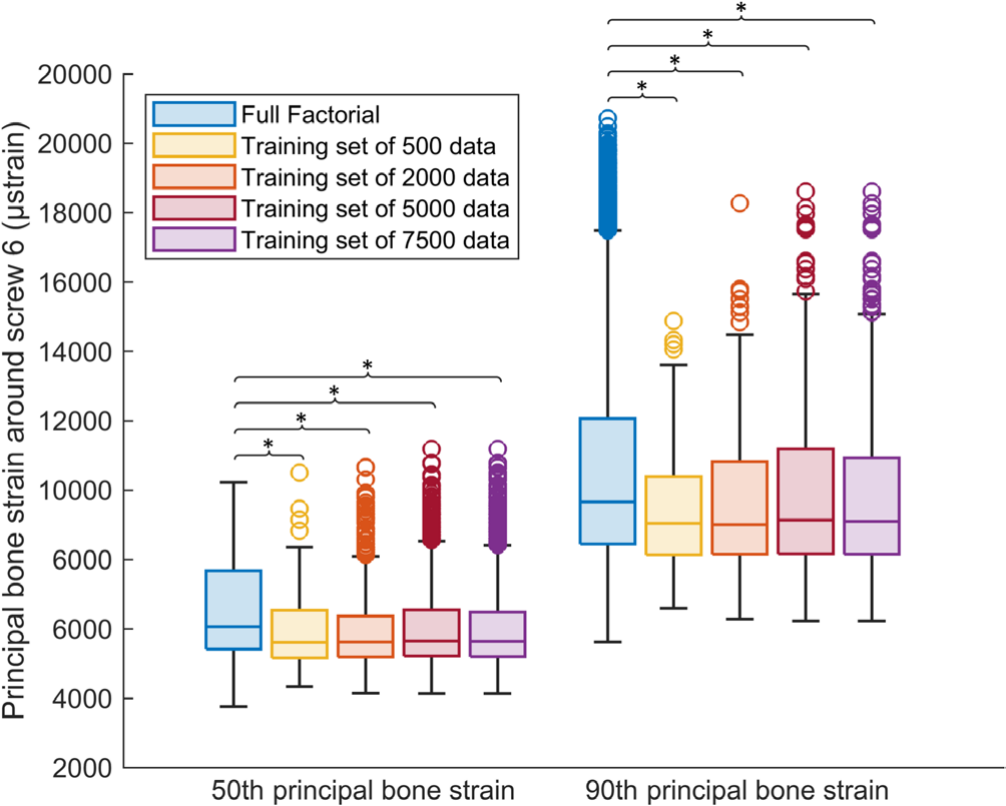
Variation of 50th and 90th percentile bone principal strain for the ANN predictions of the full factorial scenario and the FE predictions of the training set of 500, 2000, 5000, and 7500 data. Statistical significance (*p* < 0.05) is shown.

As the calcar screw has a significant impact on the biomechanical outcomes of fracture fixation plates, a final comparison between the variation of the 50th and 90th percentile principal bone strain around the calcar screw (Screw 6) predicted with the use of the ANN_collision_ with ANN_strain_16_, and the variation of the same output predicted by the FE models for the training sets of 500, 2000, 5000 was conducted. There was a statistical difference (*p* < 0.05) between the results from each of the training sets and the full factorial results (Figure 5), which show a higher range of bone principal strain.

## 4 Discussion

The aim of this study was to develop a surrogate model, using FE data, in order to make efficient predictions of bone strain of a fractured humerus with a fracture fixation plate as a result of varying the orientation of the proximal screws. A new generation of fracture fixation plates with variable angle screws has been introduced in order to improve the outcome of fracture healing, but a worst or optimal configuration hasn't been identified yet when changing the orientation of the proximal screws. Several FE studies have been conducted to investigate the impact of variable angle screws on the biomechanical performance of the fracture fixation plate, but they explored only a reduced number of configurations [12, 14, 15], or they investigated the impact of the variable angle of only two proximal screws. Indeed, conducting a FE analysis on all possible configurations is not feasible due to the high number of possible combinations. A methodology combining FE data with ANN has been developed, with the aim of making accurate and faster predictions of bone strain around the screws, which had been shown to be a screw cutout failure predictor [33]. In a previous study, we developed a similar methodology to investigate the impact of variations in screw length on the humeral strain, and our findings confirmed the efficacy of using an ANN approach trained with FE data [21]. For this particular biomechanical problem, two ANNs have been developed, one for the prediction of screw collision and another one for the prediction of screw strain. The ANN_collision_ had a maximum accuracy of 84.4%, which did not improve significantly with the increase in training set size. Two different training networks were developed for the prediction of principal bone strain, one having 14 input data (information of screw orientation) and the other having 16 input data (information of screw orientation and information of screw collision). ANN_strain_16_ showed a higher level of accuracy for the prediction of the unseen data from the testing set, especially on the prediction of the minimal principal strain of the bone around each single screw. Tables 2, 3, 4 showing that the information of screw collision used as input improves the prediction of bone principal strain while the screw orientation is varied. Overall, the ANN_strain_16_ showed to be an accurate model for the prediction of bone principal strain around the screws.

Regarding the prediction of the full factorial scenario, a purely FE approach would have taken an impractical 66,430 CPU days, with each single simulation lasting a maximum of 20 min. However, combining FE analysis and ANN considerably reduced the running time. Specifically, to produce the full factorial analysis the ANN was trained with 7500 FE simulations, which only took 18.7 CPU days. Moreover, training the ANN models and using them thereafter only took a few minutes.

Predictions of a full factorial scenario of 9^7^ simulations were made using ANN_collision_ with ANN_strain_16_, in order to assess the influence of screw orientation on bone principal strain on the full factorial spectrum. The bone principal strain predicted with ANN_collision_ with ANN_strain_16_ was not significantly different to the FE data of the unseen testing set, contrarily with the use of ANN_strain_14_, which underestimated the same data. This showed that, even the ANN_collision_ didn't have a strong level of accuracy, once combined with ANN_strain_16_ overall they were able to make similar predictions of bone principal strain compared to the FE unseen results.

The variation in the orientation of the calcar screw, Screw 6, showed to have the strongest influence on the 50th and 90th percentile of principal bone strain around all the screws (Figures 2, 3). Analyzing the 90th percentile of strain around all the screws, on average the distal position showed to be the least safe. When the Screw 6 has a distal angle of 10°, the strain value increases up to 36% from the neutral position. In terms of the safest configuration, our findings agreed with those of Fletcher et al. and Jabran et al., which identified the safest configuration was when the calcar screw is orientated in the proximal direction [12, 14]. Moreover, the impact of variations of Screw 6 on the strain of the bone around itself has been reported, showing on average a decrease of more than 20% of the variation of 50th and 90th percentile principal bone strain in the proximal direction (Figure 4). Considering the influence of varying orientations of the other screws, we observed a notable decrease in the 90th principal strain around all screws, specifically for Screw 7 when it was positioned in a more anterior location and for screw 8 when it was situated in a distal‐anterior orientation. While the other screws indicated some statistically significant improvements, the enhancements were not substantial enough, therefore considered minor.

In this study, we also compared the bone principal strain predictions obtained from the full factorial scenario generated with the ANNs with those of the FE simulations on the training sets of 500, 2000, 5000, and 7500. The analysis revealed a significant statistical difference. In particular, the ANN models were able to make predictions on the full factorial scenario with higher principal bone strain variation than the ones predicted by the FE analysis on all the training sets (Figure 5). This showed how running a full factorial analysis with an ANN approach would help to identify potentially dangerous configurations that would not have been detected through a small sample analysis.

This study has demonstrated that an FE‐informed Neural Network approach can be used to explore the impact of variation in screw orientation on bone deformation for a fracture fixation plate for a proximal humeral fracture, showing the high impact that the calcar screw orientation has on the prediction of bone principal strain. This technique has the primary advantage of being cost‐effective in terms of time, both during the training phase and when making predictions for unseen scenarios. The computational cost is strongly dependent on the generation of data used for the training process. Moreover, the process of generating new predictions was made easy and fast by utilizing only screw orientation and collision information as input data, eliminating the need for generating a new mesh for the prediction of new data. Indeed, the most cost‐demanding stage of this methodology is the generation of FE data, but this cost is significantly reduced once the surrogate model has been trained.

There are some limitations in this study. Firstly, the boundary and loading conditions of the FE analysis were simplified, no sensitivity analysis was conducted, and no muscular forces were taken into account. Incorporating muscular forces could enhance the overall strain predictions made by the FE model, resulting in more accurate outputs for the ANN models. However, adding muscular forces may also raise the computational costs associated with generating FE data, thereby raising the overall computational time. Regarding the material properties of the bone, the Morgan's equation referred to the femoral head was used to define the relation between Young's Modulus and BMD, as no equation referred to the humeral head was found in the literature [28]. The model was not experimentally validated, however, the methodology developed for the generation of the FE models reproduced in the Varga et al. study and Mischler et al., that were experimentally validated [13, 33], which was similar to the methodology conducted in this paper for the generation of FE data. In a future study, we should implement an experimental setup that aligns with the loading conditions reported by Varga et al. to achieve experimental validation of the model. In particular, use the FE results to highlight the best and worst case scenarios and replicate these experimentally in order to evaluate the ability of the model to discriminate between screw configurations This study didn't explore subject variabilities; indeed, the model was developed on a single subject, as the Jabran and Tilton studies, not taking into account the possible variations due to different anatomies [6, 11]. Since Schader et al. study [15] suggested that subject‐specific optimization of the orientation of the screws could improve the biomechanical performance of the fracture fixation plate, in the future ANN models assessing patients' variabilities should be developed. In particular, we expect significant variation in strain levels that will mostly depend on an individual's bone mineral density. This observation highlights the importance to concentrate this research on individuals with osteoporosis, particularly among women aged 65 and older, as they are more incline to undergo a surgery that involve the use of fracture fixation plates for proximal humeral fractures. Moreover, the model only included one fracture pattern in order to simplify the study. To improve the model's complexity and applicability, future versions need to take into account different fracture configurations. Another limitation of our study is the 5° increment in screw orientation, and a smaller incrementation of orientation should be implemented to achieve a better sensitivity analysis. However, we selected a 5‐degree increment for the generation of the FE data to maintain consistency with Mischler's study. When developing a full factorial scenario using the trained ANN models, we opted for 10‐degree increments due to the high computational cost involved. Given that the accuracy of the ANN_collision_ only reached 84.4%, additional techniques should be implemented and evaluated to predict screw collisions more accurately. For instance, exploring different ANN models and evaluating the potential benefits of incorporating additional data into the training process should be considered. Since the cause of the failure of these implants is unclear, the influence of other parameters should be investigated with this methodology, fracture pattern, screw configuration and also potential implant malposition. Additionally, the ANN model should include other outputs of interest to better understand the biomechanics of the fracture fixation plate, such as bone micromotion, fracture gap movements, and implant stress [7].

Despite these limitations, this study demonstrated the potential of using a finite element informed Neural Network technique to develop an advanced computational model for investigating the variation of strain with the variation of implant screws orientation. Moreover, the technique proved to be a more efficient and less time‐consuming approach than traditional methods. As also demonstrated in our previous research, using an ANN methodology is advantageous when studying problems with a wide solution space, and this approach allows for the identification of potentially dangerous configurations in a more computationally efficient manner. Our study's findings provide assurance regarding the use of this more efficient computational technique, and in the future, we intend to explore even more complex techniques to implement and potentially provide more informed decisions in a surgical setting. Specifically, future implementations should concentrate on applying the techniques developed in this study to a larger group of subjects, in order to address variations in bone mineral density and fracture patterns.

## 5 Conclusion

In conclusion, a computational approach using FE and ANNs to predict bone deformation of the humerus with the variation of screw orientation was successfully developed. This methodology showed good accuracy for the prediction of deformation of the bone with the variation of screw orientation in the proximal‐distal and anterior–posterior direction. The trained ANNs demonstrated the impact of the orientation of the calcar screws on the biomechanical performance of the fracture fixation plate, in agreement with what was found in the literature, showing that more beneficial configurations can be reached with variable angle locking screws.

## Author Contributions

D.M. designed the study, performed the numerical calculations, analyzed the data and wrote the manuscript. K.J.R. and M.T. made contributions to the study concept and design, data analysis and interpretation, editing, and revising the manuscript. All authors have read and approved the submitted version.

## Ethics Statement

The authors have nothing to report.

## Conflicts of Interest

The authors declare no conflicts of interest. The authors had permission to use the CT image from the Free Access Decedent Database funded by the National Institute of Justice grant number 2016‐DN‐BX‐0144.

## Data Availability

Research data are not shared.

## References

[1] S. M. Sporer , J. N. Weinstein , and K. J. Koval , “The Geographic Incidence and Treatment Variation of Common Fractures of Elderly Patients,” Journal of the American Academy of Orthopaedic Surgeons 14, no. 4 (2006): 246–255. 16585366 10.5435/00124635-200604000-00006

[2] A. S. McLean , N. Price , S. Graves , A. Hatton , and F. J. Taylor , “Nationwide Trends in Management of Proximal Humeral Fractures: An Analysis of 77,966 Cases From 2008 to 2017,” Journal of Shoulder and Elbow Surgery 28, no. 11 (2019): 2072–2078, 10.1016/j.jse.2019.03.034. 31420225

[3] M. Palvanen , P. Kannus , S. Niemi , and J. Parkkari , “Update in the Epidemiology of Proximal Humeral Fractures,” Clinical Orthopaedics and Related Research 442 (2006): 87–92, 10.1097/01.blo.0000194672.79634.78. 16394745

[4] F. Kralinger , M. Blauth , J. Goldhahn , et al., “The Influence of Local Bone Density on the Outcome of One Hundred and Fifty Proximal Humeral Fractures Treated With a Locking Plate,” Journal of Bone and Joint Surgery 96, no. 12 (2014): 1026–1032, 10.2106/JBJS.M.00028. 24951739

[5] P. Cronier , G. Pietu , C. Dujardin , N. Bigorre , F. Ducellier , and R. Gerard , “The Concept of Locking Plates,” Orthopaedics & Traumatology, Surgery & Research 96, no. 4 SUPPL (2010): S17–S36, 10.1016/j.otsr.2010.03.008. 20447888

[6] A. Jabran , C. Peach , and L. Ren , “Biomechanical Analysis of Plate Systems for Proximal Humerus Fractures: A Systematic Literature Review,” Biomedical Engineering Online 17, no. 1 (2018): 1–30, 10.1186/s12938-018-0479-3. 29703261 PMC5923007

[7] G. S. Lewis , D. Mischler , H. Wee , J. S. Reid , and P. Varga , “Finite Element Analysis of Fracture Fixation,” Current Osteoporosis Reports 19, no. 4 (2021): 403–416, 10.1007/s11914-021-00690-y. 34185266 PMC8422380

[8] J. W. A. Fletcher , M. Windolf , R. G. Richards , B. Gueorguiev , J. Buschbaum , and P. Varga , “Importance of Locking Plate Positioning in Proximal Humeral Fractures as Predicted by Computer Simulations,” Journal of Orthopaedic Research 37, no. 4 (2019): 957–964, 10.1002/jor.24235. 30690786

[9] J. W. A. Fletcher , M. Windolf , L. Grünwald , R. G. Richards , B. Gueorguiev , and P. Varga , “The Influence of Screw Length on Predicted Cut‐Out Failures for Proximal Humeral Fracture Fixations Predicted by Finite Element Simulations,” Archives of Orthopaedic and Trauma Surgery 139, no. 8 (2019): 1069–1074, 10.1007/s00402-019-03175-x. 30895465

[10] J. W. A. Fletcher , M. Windolf , R. G. Richards , B. Gueorguiev , and P. Varga , “Screw Configuration in Proximal Humerus Plating has a Significant Impact on Fixation Failure Risk Predicted by Finite Element Models,” Journal of Shoulder and Elbow Surgery 28, no. 9 (2019): 1816–1823, 10.1016/j.jse.2019.02.013. 31036421

[11] M. Tilton , A. D. Armstrong , H. Wee , M. W. Hast , G. Manogharan , and G. S. Lewis , “Finite Element‐Predicted Effects of Screw Configuration in Proximal Humerus Fracture Fixation,” Journal of Biomechanical Engineering 142, no. 8 (2020): 1–7, 10.1115/1.4045907. 31913444

[12] A. Jabran , C. Peach , Z. Zou , and L. Ren , “Parametric Design Optimisation of Proximal Humerus Plates Based on Finite Element Method,” Annals of Biomedical Engineering 47, no. 2 (2019): 601–614, 10.1007/s10439-018-02160-6. 30386950 PMC6342901

[13] D. Mischler , J. F. Schader , J. Dauwe , and P. Varga , “Locking Plates With Computationally Enhanced Screw Trajectories Provide Superior Biomechanical Fixation Stability of Complex Proximal Humerus Fractures,” Frontiers in Bioengineering and Biotechnology 10 (2022): 1–11, 10.3389/fbioe.2022.919721. PMC9260250 35814016

[14] D. Mischler , M. Windolf , B. Gueorguiev , S. Nijs , and P. Varga , “Computational Optimisation of Screw Orientations for Improved Locking Plate Fixation of Proximal Humerus Fractures,” Journal of Orthopaedic Translation 25 (2020): 96–104, 10.1016/j.jot.2020.02.007.

[15] J. F. Schader , D. Mischler , J. Dauwe , R. G. Richards , B. Gueorguiev , and P. Varga , “One Size May Not Fit All: Patient‐Specific Computational Optimization of Locking Plates for Improved Proximal Humerus Fracture Fixation,” Journal of Shoulder and Elbow Surgery (2021): 192–200, 10.1016/j.jse.2021.06.012. 34298147

[16] P. Varga , J. A. Inzana , J. W. A. Fletcher , et al., “Cement Augmentation of Calcar Screws May Provide the Greatest Reduction in Predicted Screw Cut‐Out Risk for Proximal Humerus Plating Based on Validated Parametric Computational Modelling,” Bone and Joint Research 9, no. 9 (2020): 534–542, 10.1302/2046-3758.99.BJR-2020-0053.R1. 32922762 PMC7469511

[17] P. Varga , J. A. Inzana , B. Gueorguiev , N. P. Südkamp , and M. Windolf , “Validated Computational Framework for Efficient Systematic Evaluation of Osteoporotic Fracture Fixation in the Proximal Humerus,” Medical Engineering and Physics 57 (2018): 29–39, 10.1016/j.medengphy.2018.04.011. 29728330

[18] Y. Jin , “A Comprehensive Survey of Fitness Approximation in Evolutionary Computation,” Soft Computing 9, no. 1 (2005): 3–12, 10.1007/s00500-003-0328-5.

[19] D. O'Rourke , S. Martelli , M. Bottema , and M. Taylor , “A Computational Efficient Method to Assess the Sensitivity of Finite‐Element Models: An Illustration With the Hemipelvis,” Journal of Biomechanical Engineering 138, no. 12 (2016): 121008, 10.1115/1.4034831. 27685017

[20] W. Takian , S. Rooppakhun , A. Ariyarit , and S. Sucharitpwatskul , “Optimal Conformity Design of Tibial Insert Component Based on ISO Standard Wear Test Using Finite Element Analysis and Surrogate Model,” Symmetry 13, no. 12 (2021): 2377, 10.3390/sym13122377.

[21] D. Mini , K. J. Reynolds , and M. Taylor , “Assessing Screw Length Impact on Bone Strain in Proximal Humerus Fracture Fixation via Surrogate Modelling,” International Journal for Numerical Methods in Biomedical Engineering 40, no. 8 (2024): 1–17, 10.1002/cnm.3840. 38866503

[22] M. Taylor , E. Perilli , and S. Martelli , “Development of a Surrogate Model Based on Patient Weight, Bone Mass and Geometry to Predict Femoral Neck Strains and Fracture Loads,” Journal of Biomechanics 55 (2017): 121–127, 10.1016/j.jbiomech.2017.02.022. 28325584

[23] R. M. A. Al‐Dirini , S. Martelli , and M. Taylor , “Computational Efficient Method for Assessing the Influence of Surgical Variability on Primary Stability of a Contemporary Femoral Stem in a Cohort of Subjects,” Biomechanics and Modeling in Mechanobiology 19, no. 4 (2020): 1283–1295, 10.1007/s10237-019-01235-0. 31637534

[24] M. T. Bah , P. B. Nair , M. Taylor , and M. Browne , “Efficient Computational Method for Assessing the Effects of Implant Positioning in Cementless Total Hip Replacements,” Journal of Biomechanics 44, no. 7 (2011): 1417–1422, 10.1016/j.jbiomech.2010.12.027. 21295306

[25] H. Edgar , S. Daneshvari Berry , E. Moes , N. Adolphi , P. Bridges , and K. Nolte , New Mexico Decedent Image Database (University of New Mexico, 2020), 10.25827/5s8c-n515.

[26] Foundation AO , “Müller AO Classification of Fractures—Long Bones,” (2014).

[27] F. Eggermont , N. Verdonschot , Y. van der Linden , and E. Tanck , “Calibration With or Without Phantom for Fracture Risk Prediction in Cancer Patients With Femoral Bone Metastases Using CT‐Based Finite Element Models,” PLoS One 14, no. 7 (2019): 1–13, 10.1371/journal.pone.0220564. PMC6667162 31361790

[28] E. F. Morgan , H. H. Bayraktar , and T. M. Keaveny , “Trabecular Bone Modulus‐Density Relationships Depend on Anatomic Site,” Journal of Biomechanics 36, no. 7 (2003): 897–904, 10.1016/S0021-9290(03)00071-X. 12757797

[29] L. Kamer , H. Noser , A. W. Popp , M. Lenz , and M. Blauth , “Computational Anatomy of the Proximal Humerus: An Ex Vivo High‐Resolution Peripheral Quantitative Computed Tomography Study,” Journal of Orthopaedic Translation 4 (2016): 46–56, 10.1016/j.jot.2015.09.006. 30035065 PMC5987007

[30] D. Krappinger , T. Roth , M. Gschwentner , et al., “Preoperative Assessment of the Cancellous Bone Mineral Density of the Proximal Humerus Using CT Data,” Skeletal Radiology 41, no. 3 (2012): 304, 10.1007/s00256-011-1174-7. 21509434

[31] G. Röderer , S. Brianza , D. Schiuma , and A. Tami , “Mechanical Assessment of Local Bone Quality to Predict Failure of Locked Plating in a Proximal Humerus Fracture Model,” Orthopedics 36, no. 9 (2013): 1134–1140, 10.3928/01477447-20130821-14. 24025003

[32] G. Bergmann , F. Graichen , A. Bender , M. Kääb , A. Rohlmann , and P. Westerhoff , “In Vivo Glenohumeral Contact Forces‐Measurements in the First Patient 7 Months Postoperatively,” Journal of Biomechanics 40, no. 10 (2007): 2139–2149, 10.1016/j.jbiomech.2006.10.037. 17169364

[33] P. Varga , L. Grünwald , J. A. Inzana , and M. Windolf , “Fatigue Failure of Plated Osteoporotic Proximal Humerus Fractures Is Predicted by the Strain Around the Proximal Screws,” Journal of the Mechanical Behavior of Biomedical Materials 75 (2017): 68–74, 10.1016/j.jmbbm.2017.07.004. 28697401

